# Aberrant alternative splicing in breast cancer

**DOI:** 10.1093/jmcb/mjz033

**Published:** 2019-05-08

**Authors:** Quan Yang, Jinyao Zhao, Wenjing Zhang, Dan Chen, Yang Wang

**Affiliations:** 1 Institute of Cancer Stem Cell, Dalian Medical University, Dalian 116044, China; 2 Department of Pathology, First Affiliated Hospital, Dalian Medical University, Dalian 116044, China

**Keywords:** alternative splicing, breast cancer, splicing factors, drug resistance, cancer therapeutics

## Abstract

Alternative splicing is critical for human gene expression regulation, which plays a determined role in expanding the diversity of functional proteins. Importantly, alternative splicing is a hallmark of cancer and a potential target for cancer therapeutics. Based on the statistical data, breast cancer is one of the top leading causes of cancer-related deaths in women worldwide. Strikingly, alternative splicing is closely associated with breast cancer development. Here, we seek to provide a general review of the relationship between alternative splicing and breast cancer. We introduce the process of alternative splicing and its regulatory role in cancers. In addition, we highlight the functions of aberrant alternative splicing and mutations of splicing factors in breast cancer progression. Moreover, we discuss the role of alternative splicing in cancer drug resistance and the potential of being targets for cancer therapeutics.

## Introduction

RNA splicing is a form of RNA processing in which a newly made precursor messenger RNA (pre-mRNA) transcript is transformed into a mature messenger RNA (mRNA) and pre-mRNAs become mature mRNAs via the excision of introns and ligation of exons during this process (Ladomery, 2013). RNA splicing takes place within the nucleus either during or immediately after transcription in nearly all mammalian cells (Han et al., 2011; Wang et al., 2013). Growing evidence demonstrates that widespread alternative splicing has been the main source of protein diversity in >90% of human genes, which has become one of the important molecular markers of human cancer and potential target for the development of new cancer therapeutics (Ladomery, 2013; Sveen et al., 2016).

Global analysis has discovered at least >15000 cancer-specific splice variants in 27 types of cancers (He et al., 2009; Kahles et al., 2018). Importantly, aberrant alternative splicing causes many human diseases including cancer, especially breast cancer. Moreover, deregulated splicing is involved in the biogenesis and progression of tumors, including cell proliferation, apoptosis, invasion, tumor metastasis, angiogenesis, and chemo/radiotherapeutic resistance (Martínez-Montiel et al., 2015; Sebestyen et al., 2015). For example, the key apoptotic regulatory gene Bcl-x could be spliced into two isoforms with opposite functions in regulating apoptosis. The short isoform Bcl-xS promotes apoptosis, whereas the long isoform Bcl-xL suppresses apoptosis. Thus, overexpression of Bcl-xL is associated with an increased risk of metastasis in breast cancer (Mercatante et al., 2001). In addition, cell surface molecule CD44 contains nine variable exons between its constitutive exons, which account for the generation of >20 splice variants (Brown et al., 2011). The inclusion of one or more of the variable exons generates CD44 variant isoforms (CD44v), while skipping all of the variable exons produces CD44 standard isoform (CD44s) (Brown et al., 2011). Generally, CD44v is expressed in epithelial cells, while CD44s is mainly expressed in mesenchymal cells. Therefore, the splicing switch from CD44v to CD44s is essential for epithelial-to-mesenchymal transition (EMT) and breast cancer metastasis (Brown et al., 2011; Xu et al., 2014; Zhang et al., 2019).

Breast cancer is one of the most common malignancies in women. According to 2018 cancer statistics, the three most common cancers for women are breast, lung, and colorectal cancers, which collectively represent one-half of all cancer cases (Siegel et al., 2018). Breast cancer accounts for 30% of all new cancers diagnosed and is the second global leading cause of cancer deaths in women (Siegel et al., 2018). The factors that contribute to the international variation in incidence rates are largely due to differences in reproduction, family history, genetic factors, and environmental factors (Hulka and Moorman, 2001; Venables et al., 2008; Jemal et al., 2011). Although the occurrence rate of breast cancer has dropped in recent years, it is still one of the most common death threats for women worldwide (Gyawali et al., 2016; Siegel et al., 2018).

Along with the finding of estrogen receptor (ER), progesterone receptor (PR), and human epidermal growth factor receptor (HER2), breast cancer subtypes were defined as luminal A-like (ER+PR+HER2−), luminal B-like (ER+PR−HER2− or ER+PR+/PR−HER2+), HER2-positive (ER−PR−HER2+), triple-negative (ER−PR−HER2−), and normal-like tumors, which are biologically variable in gene expression, phenotype, response to treatment, and outcomes that might be associated with distinct etiology (Britton, 2002; Sotiriou et al., 2003; Sorlie, 2004; Ma et al., 2006; Anderson et al., 2014; Barnard et al., 2015; Ellingjord-Dale et al., 2017). It is well established that different breast cancer subtypes have distinct prognoses and responses to chemoprevention and chemotherapy. The diverse natural history of each subtype suggests that breast cancer subtypes might also have unique risk profiles (Barnard et al., 2015). Intriguingly, aberrant splicing of genomic loci for ER and HER2 have been shown to contribute to breast carcinogenesis, which could be a potential target for cancer therapy (Basaran et al., 2018; Ponde et al., 2018). Meanwhile, aberrant alternative splicing has been revealed to be one of the important risk profiles in breast cancer.

## The regulatory process of alternative splicing

Alternative splicing is one of the most prevalent mechanisms of gene regulation. The mechanisms of alternative splicing are far more complex than the constitutive splicing. During constitutive splicing, every intron is removed and every exon is joined together to form a mature mRNA (Pozzoli and Sironi, 2005). However, alternative splicing is the process of the combinatorial rearrangement of exons, parts of exons, and/or even parts of introns into mature RNA to result in a multitude of transcripts and proteome diversity (Ast, 2004; Bates et al., 2017). Accordingly, there are five major types of alternative splicing: exon skipping also known as cassette exon, mutually exclusive exons, alternative 5′ or 3′ splice site, and intron retention (Ast, 2004). Alternative splicing has been reported to be analogous to transcriptional regulation in determining tissue- and species-specific differentiation patterns and the etiology of hereditary disease and cancer (Kornblihtt et al., 2013; Naftelberg et al., 2015).

With the rapid development in technologies in gene expression profiling, we currently have much more comprehensive knowledge about the entire collection of different transcripts encoded by the human genome. And with the confluence of whole-exome sequencing paired with RNA-seq and The Cancer Genome Atlas (TCGA) data, we can comprehensively study splicing across cancers (Hoyos and Abdel-Wahab, 2018). The alternative splicing process, in which different combinations of distinct regions of pre-mRNAs are selected to form different mature mRNAs. This process is one of the most robust mechanisms to achieve genetic diversity (Matlin et al., 2005; Kalsotra and Cooper, 2011). Many aberrant splicing events of cancer-related genes have been discovered in a variety of cancers, which are critical for tumorigenesis and cancer metastasis. Currently, aberrant splicing is systematically investigated not only for the underlying mechanisms but also as potential biomarkers for diagnosis and therapeutic development in cancer (Zhang and Manley, 2013).

**Figure 1:**
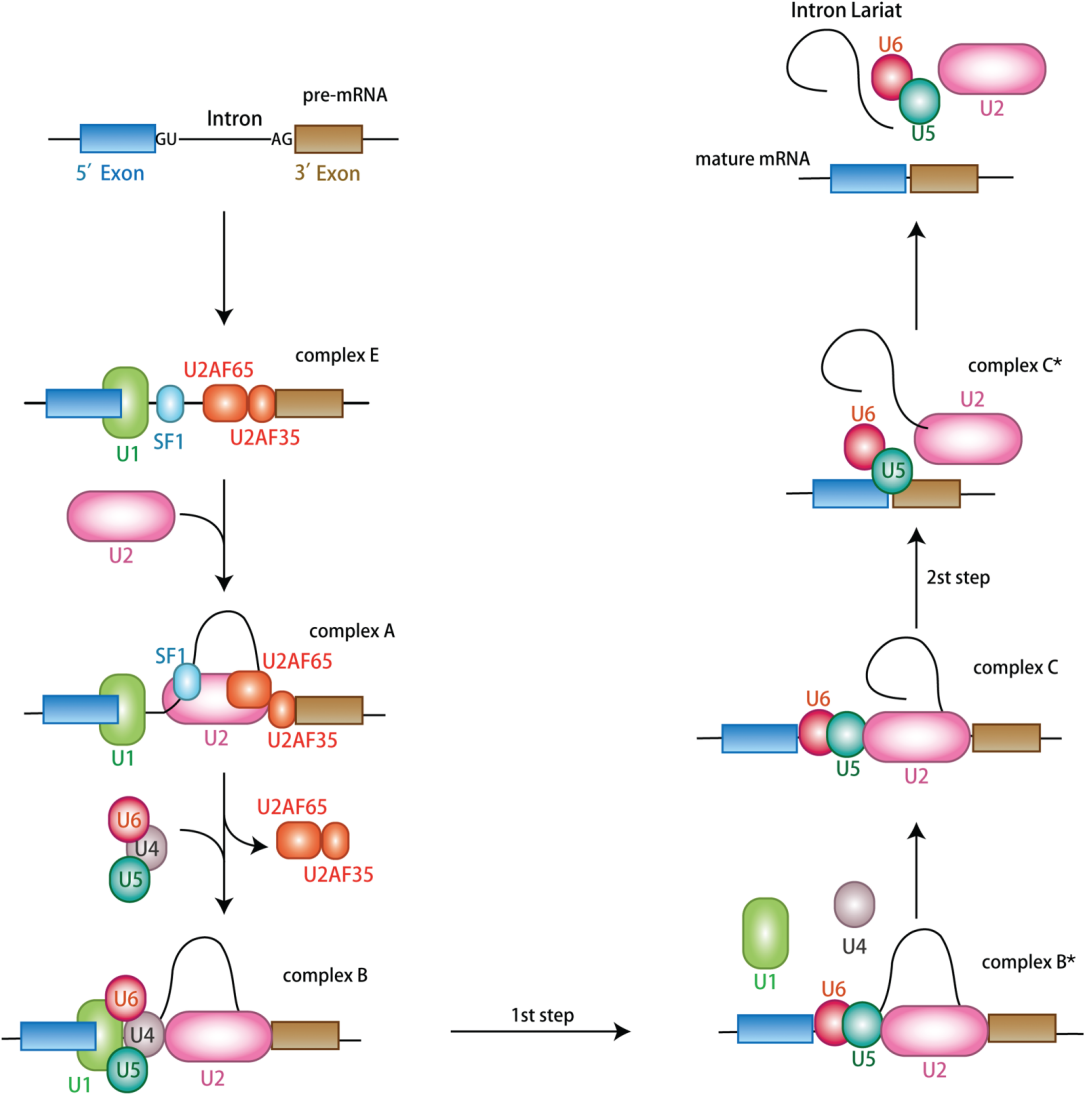
Pre-mRNA splicing process catalyzed by major spliceosome. Splicing occurs in several steps with the assembly of spliceosomes. The U1, U2, U4/U6, and U5 snRNPs are the major components of spliceosomes, and each snRNP comprises a small nuclear RNA and various associated proteins. Splicing begins with the binding of U1 snRNP to the intronic 5′ splice site, which is ATP-independent. Subsequently, this reaction is stabilized by the binding of SF1 and splicing factor U2AF65 to the 3′ splice site, forming the early complex E. Consequently, the ATP-dependent recruitment of U2 snRNP to the intron BPS, thereby forming the pre-spliceosome (complex A). The pre-assembled U4–U6–U5 tri-snRNP is recruited to the pre-spliceosome to generate complex B. This reaction undergoes a series of complex changes, including the release of U1 and U4, forming the catalytically active complex B (complex B^*^), which is involved in the first catalytic step of splicing (complex C). The resulting complex undergoes further rearrangements for the second catalytic step of splicing. Finally, U2, U5, and U6 snRNPs are released to form mature mRNA.

The splicing process undergoes sequential phosphodiester transfer reactions, which is catalyzed by large ribonucleoprotein complexes known as spliceosomes, including the small nuclear ribonucleoproteins (snRNPs) U1, U2, U4, U5, and U6 and splicing factors (Grabowski et al., 1985; Cartegni, 2003; Wang and Burge, 2008; Valadkhan, 2014). Spliceosomes are assembled stepwise at the intron/exon junctions of precursor RNAs known as splice sites (Naftelberg et al., 2015). The 5′ splice site is present at the initiation of an intron, and the 3′ splice site is located at the end of an intron, whereas the branch point sequence (BPS) is usually located at ~15–50 nucleotides upstream of the 3′ splice site. Splice sites can be strong or weak depending on how far their sequences diverge from the consensus sequence (Kornblihtt et al., 2013). Splicing begins with a weak interaction of U1 snRNP with the 5′ splice site, which is adenosine triphosphate (ATP)-independent, and subsequently stabilized by the binding of splicing factor 1 (SF1) and U2AF65 to the 3′ splice site. Together, these structures form the early complex E and trigger the ATP-dependent recruitment of U2 snRNP to the intron branch point, thereby forming the pre-spliceosome (complex A). This reaction also brings the 5′ splice site, branch point, and 3′ splice site into closer proximity. Then, the pre-assembled U4–U5–U6 snRNP complex will be recruited to the pre-spliceosome and then release U1 and U4 snRNPs to form a catalytically active complex B^*^, which is also a part of the first catalytic step of splicing. Next, the complex containing the free end of the first exon and the remaining intron–exon lariat intermediate will carry out a series of rearrangements to prepare for the second catalytic step in an ATP-dependent manner. Finally, the U2, U5, and U6 snRNPs will be released from the complex for the subsequent splicing reactions in order to form mature mRNAs (Figure 1) (Kornblihtt et al., 2013; Lee and Rio, 2015; Shi, 2017; Paschalis et al., 2018).

Moreover, additional information that determines the splicing specificity is also provided by multiple *cis*-regulatory elements that serve as either splicing enhancers or silencers (Naftelberg et al., 2015). According to the distinct locations and functions, these *cis*-elements are classified as exonic splicing enhancers (ESEs) or silencers (ESSs), and intronic splicing enhancers (ISEs) or silencers (ISSs) (Ast, 2004). *Cis*-elements are recruiting *trans*-acting splicing factors to activate or suppress the usage of splice sites or spliceosome assembly through various mechanisms (Fairbrother et al., 2002; Wang et al., 2013).

## Aberrant alternative splicing events in breast cancer

### Alternative splicing of breast cancer type 1

**Figure 2:**
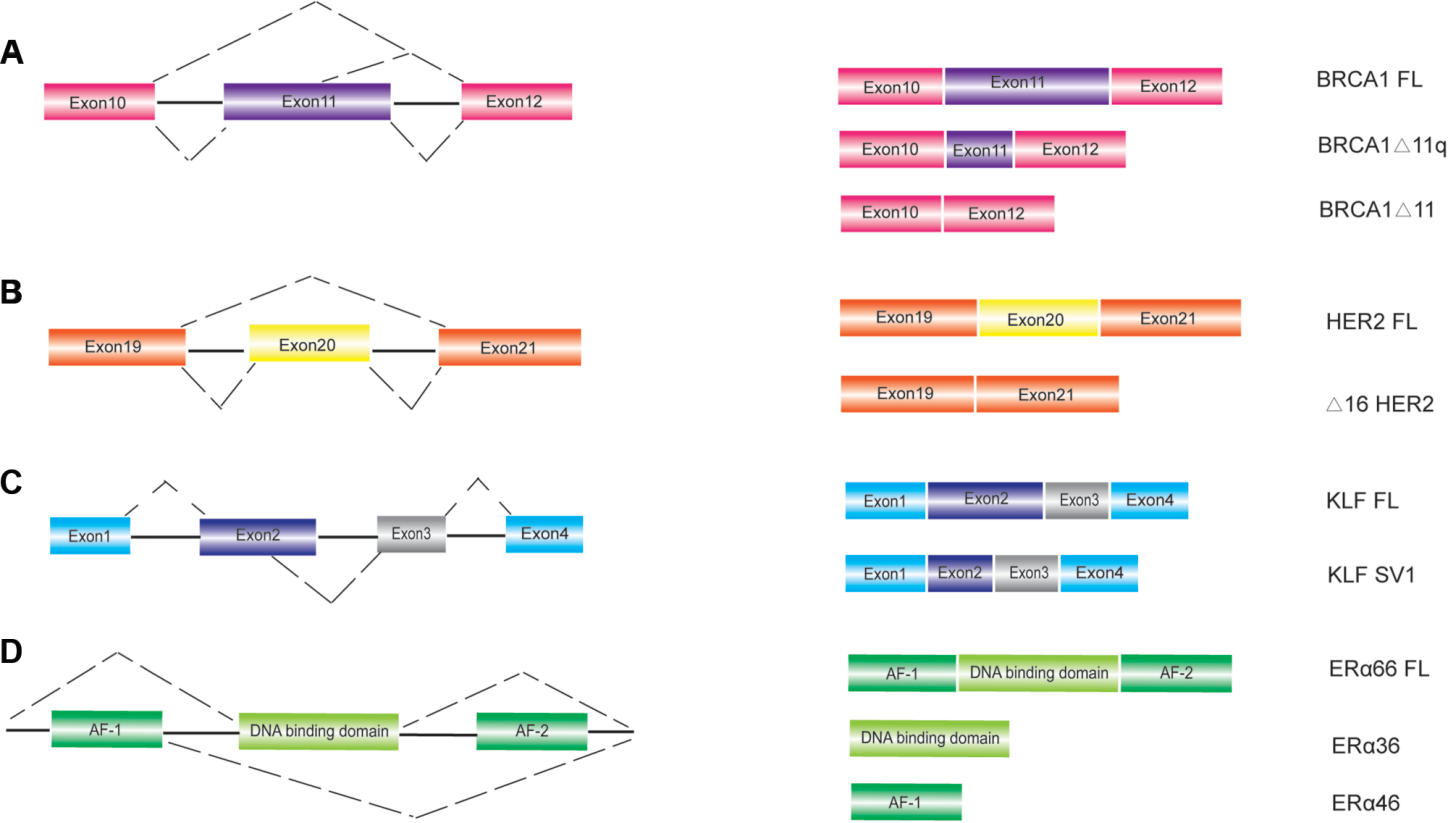
Alternative splicing events involved in breast cancer. A schematic of several important alternative splicing events in breast cancer. The pre-mRNA regions of BRCA1 (**A**), HER2 (**B**), KLF (**C**), and ERα (**D**) are demonstrated in the left, and the splice variants are shown in the right. FL represents the full-length isoform. BRCA1-Δ11q represents partial skipping of exon 11. BRCA1-Δ11 represents skipping of exon 11.

Breast cancer type 1 (BRCA1) is a tumor suppressor gene, which is involved in DNA repair by homologous recombination and interacts with different partners to maintain the genomic stability (Narod and Foulkes, 2004). In the 1990s, the BRCA1 DNA repair gene was reported to be associated with hereditary breast cancer. Subsequently, BRCA1 is proved to be major breast cancer susceptibility genes, whose pathogenic variants are significantly associated with an increased risk of breast and ovarian cancers (Miki et al., 1994; Walsh et al., 2006; Schmidt et al., 2017). Alternative spliced BRCA1 has three major isoforms that depend on the regulation of exon 11, including BRCA1 full-length (inclusion of all coding exons), BRCA1-Δ11 (skipping of exon 11), and BRCA1-Δ11q (partial skipping of exon 11) (Figure 2A). The BRCA1-Δ11q isoform derives from the usage of an alternative donor splice site in exon 11, resulting in the exclusion of most exon 11 sequences. It has been reported that breast cancer patients bearing exon 11 mutation have a worse overall survival as compared to non-exon 11 mutation carriers. In addition, BRCA1-Δ11q is also positively correlated to tumorigenesis and drug resistance (Nielsen et al., 2016; Wang et al., 2016).

### Alternative splicing of HER2

The oncogene HER2 is coding for a tyrosine kinase receptor, whose overexpression or amplification delineates an HER2-positive breast cancer subtype. It is characterized by a high mitotic index and an elevated metastatic potential, which is considered intrinsically heterogeneous, both biologically and genetically (Prat et al., 2014; Nielsen et al., 2016). Δ16HER2, a splice variant of HER2, lacks exon 20 that encodes a small extracellular region (Inoue and Fry, 2015) (Figure 2B). Importantly, emerging evidence suggests that the co-existence of the full-length/wild-type HER2 oncoprotein with Δ16HER2 significantly increases the heterogeneity of HER2-positive disease, affecting its biology, clinical course, and treatment response (Weigelt and Reis-Filho, 2013). In addition, it is also reported that Δ16HER2 initiates a key oncogenic signal that has a significant impact on HER2-driven breast cancer stemness (Castiglioni et al., 2006), tumorigenesis (Turpin et al., 2016), and drug resistance (Jackson et al., 2013) as compared to its full-length counterpart.

### Alternative splicing of Kruppel-like factor 6

Kruppel-like factor 6 (KLF6) is a tumor-suppressing protein whose expression is reduced in a majority of breast cancer patients (Liu et al., 2010; Ozdemir et al., 2014). Remarkably, KLF6 encodes multiple protein isoforms derived from alternative splicing, most of which are intimately involved in tumorigenesis and tumor progression. Three main splicing isoforms have been identified, including KLF6-SV1, KLF6-SV2, and KLF6-SV3 (DiFeo et al., 2009) (Figure 2C). The full-length KLF6 and KLF6-SV3 localize to the nucleus because of the retention of the nuclear localization signal (NLS) in exon 2. However, KLF6-SV1 and KLF6-SV2 usually localize to cytoplasm due to the absence of NLS, thereby promoting breast cancer cell proliferation and breast cancer metastasis. In addition, KLF6-SV1 is also a key driver of breast cancer metastasis, thus to provide great therapeutic potentials for invasive breast cancer (Hatami et al., 2013; Liang et al., 2014).

### Alternative splicing of ERα and ERβ

ER alpha (ERα) gene produces various isoforms through alternative splicing in a tissue and disease-specific manner (Herynk and Fuqua, 2004; Taylor et al., 2010). The classic full-length ERα66 harbors two activation domains, AF-1 and AF-2. The short isoform ERα36 encodes a 29-amino acid protein, which lacks AF-1 and AF-2 domains. Another splicing isoform ERα46 only contains AF-1 domain (Chantalat et al., 2016), whose sequence is identical to the sequences from 174 to 595 amino acid of ERα66 (Inoue and Fry, 2015) (Figure 2D). Strikingly, ERα46 antagonizes the function of the full-length ERα66 in mammary carcinoma cells. In addition, ERα46 has also been reported to be involved in breast cancer development and drug resistance (Li et al., 2003; Klinge et al., 2010).

ER beta (ERβ) inhibits breast cancer cell proliferation and tumor growth. The expression level of ERβ is correlated to a better prognosis of breast cancer (Leung et al., 2012; Haldosen et al., 2014). Five ERβ isoforms have been identified, including ERβ1, ERβ2, ERβ3, ERβ4, and ERβ5. ERβ1 and ERβ2 demonstrate distinct expression levels in normal epithelial and non-epithelial parts of breast cancer cells and tissues, indicating that they play different biological roles in normal tissues and transformed cells. ERβ1 might target IRE1/XBP-1 pathway to promote the apoptosis of breast cancer cells (Rajapaksa et al., 2015). However, the disease-free survival and overall survival are poor in ERβ2-positive breast cancer patients (Baek et al., 2015).

## The regulatory role of splicing factors in cancers

Splicing factors are RNA-binding proteins that target specific RNA sequences or motifs. Once splicing factors bind to pre-mRNAs, they can both guide or block the interaction between spliceosome and pre-mRNAs, which also suggests they play dual roles in activating or inhibiting splicing. Currently, a great number of splicing factors have been identified in human cells and tissues (Long and Caceres, 2009).

Common splicing factors can be divided into two key families, including serine/arginine-rich (SR) proteins (Long and Caceres, 2009) and heterogenous ribonuclear proteins (hnRNPs). Mechanistically, SR proteins act as splicing activators by binding to the ESEs or inhibit splicing by binding introns (Dvinge et al., 2016; Howard and Sanford, 2015). HnRNPs could either positively or negatively control splicing through binding to different pre-mRNA regions (Busch and Hertel, 2012). Importantly, the expression level, localization, and mutations of splicing factors determine the splicing outcomes in distinct tissues and cells (Wang et al., 2014).

### The regulatory role of SR proteins in cancers

Twelve classical SR-rich splicing factors (SRSFs) have been identified, including SRSF1–SRSF12. SR proteins contain at least one RNA recognition motif (RRM) and a downstream arginine/serine (RS) domain. The RRM is responsible for RNA binding and the RS domain mediates protein–protein interactions (Krainer et al., 1990; Busch and Hertel, 2012).

**Figure 3:**
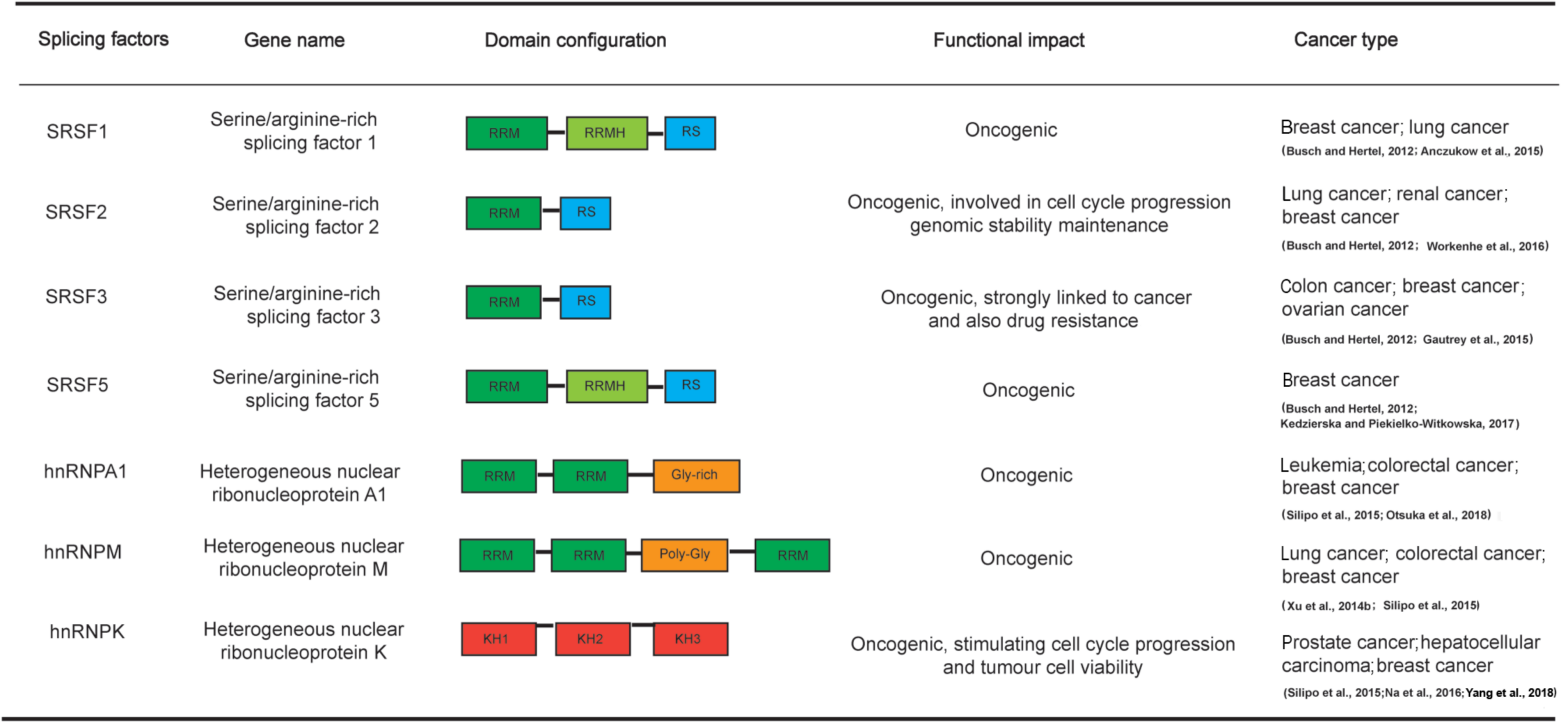
A summary of the main domain configurations of some human SR proteins (SRSF1–SRSF3, SRSF5) and hnRNP proteins (hnRNPA1, hnRNPM, hnRNPK) and their functional impacts in cancers.

Emerging evidences have demonstrated that misregulation and post-translational modification of SR proteins can play critical roles in controlling alternative splicing in cancer. Among the SR proteins, SRSF1 (Anczukow et al., 2015), SRSF2 (Workenhe et al., 2016), SRSF3 (Gautrey et al., 2015), SRSF5, and SRSF6 (Kedzierska and Piekielko-Witkowska, 2017) have been shown to be highly expressed in breast cancer (Figure 3). Significantly, with the high-throughput sequencing technologies development, mutations of spliceosome components especially SR proteins have been identified as a novel class of driver mutations in a variety of human diseases, such as cancer and myelodysplastic syndromes (MDS) (Jenkins and Kielkopf, 2017). More importantly, the frequency of mutations varies significantly across different diseases (Dvinge et al., 2016).

As reported, SF3B1, one of the most important spliceosome components, normally regulates splicing assembly by promoting alternative branch point usage. However, mutations of SF3B1 are extremely frequent in uveal melanoma and breast cancers, as well as MDS (Stephens et al., 2012; Harbour et al., 2013). Meanwhile, SRSF2 mutations are more common in chronic myelomonocytic leukemia, which alter the RNA-binding characteristics of SRSF2, thereby resulting in extensive changes in splicing patterns and impairment of hematopoietic cell differentiation (Yoshida and Ogawa, 2014; Shiozawa et al., 2018). Additionally, the oncogenic SRSF1 and SRSF3 are overexpressed in multiple human cancers, including lung, colon, and breast cancers, despite limited mutations of them have been revealed (Shilo et al., 2015). SRSF1 (previously known as SF2/ASF) has multiple biological functions, including the regulation of nonsense-mediated mRNA decay, mRNA export, and translation (Wang et al., 2014). Crucially, SRSF1 could induce aberrant splicing of MNK2 and S6K1 to activate the mTOR pathway (Malakar et al., 2017). In addition, SRSF1 could also control the splicing of many apoptotic genes, such as MCL (Scotti and Swanson, 2016), BIM (Anczukow and Krainer, 2016), and Bcl-x (Kedzierska and Piekielko-Witkowska, 2017) to regulate apoptosis in cancers.

The post-translational modifications of SR proteins also play vital roles in regulating the alternative splicing events in cancer cells. There are at least three main modifications have been linked to SR proteins, including phosphorylation, methylation, and acetylation, which are essential for controlling the activity and localization of SR proteins (Martinez-Montiel et al., 2018). Generally, SR proteins could be phosphorylated by two major classes of protein kinases: serine arginine protein kinases (SRPKs) and CDC-like kinases (CLKs), which phosphorylate SR proteins at distinct sites (Corkery et al., 2015; Bullock et al., 2016). Among the three SRPK members, SRPK1 and SRPK2 are directly associated with tumorigenesis and always upregulated in various types of cancers, including breast, pancreatic, colon, lung cancer, and so on (Hayes et al., 2007; Zhong et al., 2009; Gout et al., 2012). The CLKs family consist of four members that could phosphorylate SR proteins to regulate cancer-related alternative splicing events, such as CLK1 phosphorylates splicing factor 45 (SPF45) to induce migration and invasion of ovarian cancer cells (Ngo et al., 2005; Ninomiya et al., 2011; Silipo et al., 2015).

### The regulatory role of hnRNPs in cancers

The hnRNPs are a large family of proteins containing at least 20 members with common structural domains, which usually bind splicing silencers to influence both constitutive and alternative splicing events throughout the human genome (Busch and Hertel, 2012; Silipo et al., 2015). In general, hnRNPs are involved in the regulation of various cellular processes, such as RNA metabolism, DNA repair, telomere biogenesis, and gene expression (He et al., 2005). Additionally, emerging evidence suggests that hnRNPs may also play critical roles in cancer, especially breast cancer development and progression, such as proliferation, apoptosis, angiogenesis, and invasion (Xu et al., 2014; Silipo et al., 2015; Kedzierska and Piekielko-Witkowska, 2017). Several hnRNPs are reported to overexpress in breast cancer, including hnRNPA1, hnRNPA2, hnRNPI, hnRNPM, and hnRNPK (Xu et al., 2014; Silipo et al., 2015; Na et al., 2016; Otsuka et al., 2018; Yang et al., 2018) (Figure 3). In addition, hnRNPA1, hnRNPA2, and hnRNPI are favoring the splicing switch from PKM1 to PKM2 (David et al., 2010). PKM has two mutually exclusive exons: exon 9 and exon 10. Mutually inclusions of these exons lead to the production of two isoforms, including PKM1 that includes exon 9 but not exon 10, and PKM2, which contains exon 10 but not exon 9. Importantly, PKM2 is ubiquitously expressed in tumors; however, PKM1 is expressed in differentiated tissues, such as muscle and brain. The splicing switch toward PKM2 in tumor cells is necessary to trigger metabolic phenotype known as the Warburg effect (Christofk et al., 2008; David et al., 2010; Calabretta et al., 2016). Additionally, hnRNPA1 produces tumorigenic splice variants of RON, thereby reducing the formation of the EMT driving isoform ΔRON. Whereas, hnRNPA1 could also act as an oncoprotein to promote inclusion of exon 9 of tumor suppressor caspase-2, thus to produce the truncated anti-apoptotic isoform caspase-2S (David et al., 2010; Shilo et al., 2015). The splicing factor PTBP1 is positively correlated to the growth of various cancers and poor prognosis. Meanwhile, PTBP1 regulates the pro-inflammatory senescence-associated secretory phenotype by controlling the exon 7 skipping of EXOC7, thereby inducing inflammation-driven cancers (Xue et al., 2009; Georgilis et al., 2018). In addition, hnRNPM binds to the GC-rich domain of CD44 to promote the skipping of exon 8, thus to stimulate breast cancer metastasis (Xu et al., 2014; Shilo et al., 2015).

## Alternative splicing regulated by m^6^A modification in cancers 

Over 150 RNA modifications have been identified as posttranscriptional regulatory marks in RNAs, which regulate multiple RNA regulatory processes, including alternative splicing, export, stability, and translation regulation (Roundtree et al., 2017; Zhao et al., 2017; Yang et al., 2018). N^6^-methyladenosine (m^6^A) is one of the most prevalent modifications. With the development of high-throughput sequencing technology, transcriptome-wide profiling reveals >10000 m^6^A peaks, which have been validated in >25% of human transcripts (Yang et al., 2018). The biological function of dynamic RNA m^6^A modification is determined by the interplay between methyltransferases (‘writers’) (Ping et al., 2014), binding proteins (‘readers’) (Yang et al., 2018), and demethylases (‘erasers’) (Zhao et al., 2014), which could also regulate alternative splicing (Adhikari et al., 2016).

Currently, five human YTH domain-containing family proteins have been identified, including YTHDC1, YTHDC2, and YTHDF1–YTHDF3, which are all m^6^A readers and whose localization is critical for their functions (Xu et al., 2014). The nuclear reader YTHDC1 interacts with many splicing factors (e.g. SRSF1, SRSF3, SRSF7, SRSF9, and SRSF10) to affect their functions in regulating splicing (Adhikari et al., 2016). Specifically, YTHDC1 could promote exon inclusion by recruiting SRSF3 to block the binding site of SRSF10 at target pre-mRNAs, indicating that alternative splicing could be regulated in an m^6^A dependent manner (Adhikari et al., 2016; Xiao et al., 2016). Similarly, the methyltransferase METTL3, the demethylase fat mass and obesity-associated protein (FTO), and ALKBH5 could also play crucial roles in modulating alternative splicing (De Arras and Alper, 2013). For example, METTL3 might affect the LPS-induced inflammatory response by regulating the alternative splicing of MyD88 in human dental pulp cells (De Arras and Alper, 2013; Feng et al., 2018). In addition, the ‘eraser’ FTO, which has been previously demonstrated to be involved in obesity regulation (Fischer et al., 2009), could control the exon skipping of adipogenic regulatory factor 1 (RUNX1T1) by modulating m^6^A expression, thus to recruit SRSF2 to the splice site to influence adipogenesis (Yang et al., 2018). Moreover, the m^6^A demethylase ALKBH5 plays a vital role in splicing regulation during spermatogenesis in mice (Tang et al., 2018). Particularly, ALKBH5-dependent m^6^A erasure appears to be able to protect longer 3′-UTR transcripts from aberrant splicing in the nuclei of pachytene spermatocytes and round spermatids. Importantly, ALKBH5 is also involved in regulating tumorigenesis in several cancers, especially breast cancer (Zhang et al., 2016; Yang et al., 2018).

Although many recent studies have revealed that m^6^A modification seems to regulate the mRNA diversity by modulating alternative splicing, the detailed mechanisms are still elusive.

## Alternative splicing in cancer therapeutic resistance and target for therapy

### The role of alternative splicing in breast cancer therapeutic resistance

Nowadays, therapeutic resistance has become one of the major challenges in cancer treatment, leading to the low efficiency or failure of the treatment. Importantly, alternative splicing could significantly influence the expression levels and functions of cancer drug targets, thereby participating in therapeutic resistance regulation (Lee and Abdel-Wahab, 2016). The splicing of genes involved in apoptosis, DNA damage, and drug metabolism, could alter after chemotherapy, thus to promote cancer cell survival (Lu and Chao, 2012; Siegfried and Karni, 2018). For instance, cisplatin causes SRSF4-mediated splicing alteration to induce apoptosis. In addition, the PARP inhibitor olaparib has limited effects on breast cancer patients harboring germline mutations of BRCA1, especially the mutant with increased expression of BRCA1 exon 11 skipping (BRCA1-Δ11q) (Wang et al., 2016; Kim et al., 2017; Paschalis et al., 2018; Siegfried and Karni, 2018). Moreover, studies have shown the possible relationship between the unfolded protein response (UPR) with the endoplasmic reticulum and breast cancer drug resistance. Particularly, the human X-box binding protein-1 (XBP1), a key transcription factor, plays a critical role in breast cancer drug resistance through regulating alternative splicing in UPR stress signaling pathway (Davies et al., 2008). Additionally, overexpression of XBP1s, a splicing isoform of XBP1, leads to estrogen-independent cell growth in ER-positive breast cancer cells and increases the resistance of cancer cells to tamoxifen (Ding, 2003; Fang et al., 2004). Strikingly, STF-080310, a novel IRE1α/XBP1 inhibitor, can sensitize resistant MCF7 cancer cells to tamoxifen through specifically disrupting the splicing of XBP1 and reducing the expression level of XBP1s (Papandreou et al., 2011; Ming et al., 2015).

In addition to breast cancer, aberrant splicing also potentially participates in chemotherapeutic drug resistance of several other cancers. For example, the skipping of exon 3 or exon 4 of BIM results in chemoresistance of chronic myeloid leukemia (La Rosee and Hochhaus, 2008). The splicing modulators, such as spliceostatin A or its analog meayamycin B, could target SF3B1 to inhibit the splicing of BRAF, thus to sensitize resistant melanoma to vemurafenib (Poulikakos et al., 2011; Salton et al., 2015). Additionally, the splicing isoform ARv7 of the androgen receptor (AR) promotes enzalutamide-resistant in prostate cancer, which could be a potential target for anti-androgen therapy (Cao et al., 2014).

### Targeting aberrant splicing as a novel therapeutic approach in cancers

Given that aberrant splicing has been one of the hallmarks of cancer, development of therapeutic approaches to target splicing will be promising and powerful. Currently, many tools have been developed to manipulate splicing. Antisense oligonucleotides (ASOs), which are typically 15–25 bases in length, have been applied to target splice site or splicing *cis*-regulatory elements, to regulate splicing (DeVos and Miller, 2013). For example, ASOs can dramatically switch SMN2 splicing patterns, leading to nearly complete inclusion of exon 7 and thus significantly increasing functional SMN protein (Hua et al., 2010). Splicing-switching antisense oligonucleotides (SSOs), which is a kind of ASOs that are typically 15–30 nucleotides long, might be utilized to target a specific splicing enhancer or silencer to prevent the binding of *trans*-acting regulatory splicing factors, thereby effectively inhibiting or promoting splicing (Palhais et al., 2015; Havens and Hastings, 2016).

As reported, an SSO called oligoAB has been designed to target the splice site sequence of BRCA1, which is capable of altering BRCA1 pre-mRNA splicing through stimulating the BRCA1 exon 11 skipping and simultaneous reduction of BRCA1-FL and BRCA1-Δ11Q (partial skipping of exon 11) expression (Raponi et al., 2012; Wang et al., 2016). In combination with PARP inhibitor, oligoAB can enhance the efficacy of PARP inhibitor treatment in breast cancer cells (Smith et al., 2017). However, considering the splicing reactions occur in the nucleus where is difficult for SSOs to reach, CRISPR-Cas9 technology can be used to delete specific exons to manipulate splicing (Mohanraju et al., 2016). Beyond that, engineered artificial splicing factors (ESF) can also be designed by combining the Pumilio and FBF (PUF) domains of human Pumilio1 with a functional domain to regulate alternative splicing. The ESF has been applied to affect the splicing regulation of the apoptotic gene Bcl-x to increase the pro-apoptotic isoform Bcl-xS. Moreover, the function of this ESF has been tested in several types of cancer, including breast cancer cells (Wang et al., 2009).

Splicing factors can also be targets for cancer therapy. Splicing factor kinase inhibitors will be possible drugs or adjuvants for alternative splicing-dependent cancer therapy (Hsu et al., 2015). In addition, the activity of SR proteins is regulated by phosphorylation of their RS domains (Plocinik et al., 2011). The phosphorylation levels of SR protein are controlled by three main families of splicing kinases: CDC2-like kinases (CLKs), dual-specificity tyrosine-regulated kinases (DYRKs), and SR-rich splicing factor protein kinases (SRPKs) (Plocinik et al., 2011). Thus, these kinase inhibitors can be utilized to regulate alternative splicing in cancer (Hsu et al., 2015; Bates et al., 2017).

## Conclusions

Increasing evidence has shown that alternative splicing has significant effects on tumorigenesis and development of many cancers, including breast cancer. Therefore, targeting aberrant alternative splicing might provide a new avenue for cancer therapy. However, specific mechanisms still remain largely unknown, and future efforts are needed to uncover the existing splicing regulation, especially the investigations of oncogenic or tumor suppressing alternative splicing events and splicing factors. This will help us to better understand cancer-related alternative splicing and develop novel strategies for breast cancer treatment.
